# Complete Genome Sequence of Streptococcus agalactiae Strain 01173, Isolated from Kuwaiti Wild Fish

**DOI:** 10.1128/MRA.00674-20

**Published:** 2020-09-03

**Authors:** Morena Santi, Sharon A. Egan, James A. Leigh, David W. Verner-Jeffreys, Adam M. Blanchard

**Affiliations:** a School of Veterinary Medicine and Science, University of Nottingham, Sutton Bonington, Leicestershire, United Kingdom; b Cefas Weymouth Laboratory, Centre for Environment, Fisheries, and Aquaculture Science, Weymouth, Dorset, United Kingdom; University of Maryland School of Medicine

## Abstract

Here, we report the complete genome of piscine Streptococcus agalactiae 01173 serotype Ia, which was generated using long-read sequencing technology. The bacteria were isolated from wild fish displaying signs of streptococcosis, from a fish kill incident in Kuwait.

## ANNOUNCEMENT

Streptococcus agalactiae, a group B streptococcus, is an opportunistic Gram-positive bacterium that is chiefly associated with meningitis and septicemia in infants in the Western world (1) and is increasingly associated with adult infections, especially among the elderly or immunocompromised (2). S. agalactiae is a major pathogen of fish that manifests as meningoencephalitis, with high mortality rates being reported for tilapia (*Oreochromis* sp.), and it represents a significant threat to the aquaculture industry (3, 4). Here, we report the whole-genome sequence of S. agalactiae strain 01173 serotype Ia, multilocus sequence type 7 (ST7), isolated from infected tissue from a wild fish in Kuwait using sterile swabs and cultured on tryptone soya agar; species confirmation was performed by 16S rRNA sequencing (5). Bacteria were cultured overnight on brain heart infusion (BHI) agar (Oxoid) at 37°C. Single colonies were inoculated into BHI broth, and genomic DNA was extracted as described previously (6). Genomic DNA was sequenced using Illumina MiSeq and Oxford Nanopore Technologies (ONT) GridION X5 Mk1 sequencing (BioProject number PRJNA627590). ONT libraries were prepared using the SQK-RBK004 rapid barcoding sequencing kit (ONT) and then loaded onto a MinION FLO-MIN106 R9.4.1 flow cell (ONT). Illumina sequence libraries were created using the Illumina TruSeq library preparation kit, following the manufacturer’s guidelines, and were sequenced as 250-bp paired-end reads.

Raw data for both Illumina and Nanopore sequences were submitted for rigorous quality filtering. Default parameters were used throughout except where otherwise stated. Illumina data were parsed with Trimmomatic (v0.39) (7) to remove any adaptor contamination using a sliding-window approach to maintain an average Phred score of 30 across the read. Unpaired reads were discarded. Nanopore data were base called using Guppy (v3.2.8) with the high-accuracy model and were checked for adaptors using Porechop (v0.2.4) (https://github.com/rrwick/Porechop) with the addition of the discard_middle flag. The data were then parsed with Filtlong (v0.2.0) (https://github.com/rrwick/Filtlong) using the Illumina data as a reference and the flags min_length 1000 and keep_percent 90. This resulted in 627,824 Illumina and 6,196 Nanopore high-quality reads (*N*_50_, 17.1 kb). Trimmed and filtered reads were used as inputs for Unicycler (v0.4.8) (8) in the normal hybrid assembly mode, circularized, and rotated to *dnaA*. The resulting assembly was polished once using Nanopolish (v0.13.2) (https://github.com/jts/nanopolish). The assembled completed genome of S. agalactiae strain 01173 (GenBank accession number CP053027.1) was characterized by a single circular chromosome of 2,105,299 bp with a GC content of 35.7%. The assemblies were then annotated using PGAP (v4.11) (9), resulting in an 87.6% coding ratio with 2,024 protein-coding sequences, 21 rRNAs, 80 tRNAs, and 1 putative clustered regularly interspaced short palindromic repeat (CRISPR) sequence. S. agalactiae 01173 contained most virulence factors previously associated with the species, including sortase A (10), C5a peptidase, CAMP factor, complete capsular polysaccharide operon, and serine peptidase (11), and 7 copies of group II intron reverse transcriptase/maturase genes, which are frequently located near potential virulence genes (12). The S. agalactiae 01173 whole genome was compared to all publicly available, complete genomes for Streptococcus agalactiae (downloaded from the NCBI databases [accessed 24 March 2020]), and individual genomic sequence signatures were created using sourmash (v3.3.0) (13). The resulting hash tables were imported into R to generate heatmaps using a Jaccard similarity index cutoff value of 85%. S. agalactiae 01173 clustered with strains isolated from fish, as expected, particularly Mugilidae species, which are commonly found in the Kuwaiti Gulf. Two previously identified fish-associated genes were identified, i.e., genes for an α-galactosidase and an ABC transporter permease (14), which further accentuates the differences from S. agalactiae serotype Ia strains isolated from cases of human infection.

### Data availability.

All data have been deposited in the NCBI Sequence Read Archive (SRA). Raw sequence reads are available under accession number PRJNA627590, and the assembled genome is available under GenBank accession number CP053027.1.
